# Acid–base safety during the course of a very low-calorie-ketogenic diet

**DOI:** 10.1007/s12020-017-1405-3

**Published:** 2017-09-15

**Authors:** Diego Gomez-Arbelaez, Ana B. Crujeiras, Ana I. Castro, Albert Goday, Antonio Mas-Lorenzo, Ana Bellon, Cristina Tejera, Diego Bellido, Cristobal Galban, Ignacio Sajoux, Patricio Lopez-Jaramillo, Felipe F. Casanueva

**Affiliations:** 1 Division of Endocrinology, Department of Medicine, Complejo Hospitalario Universitario de Santiago (CHUS), Santiago de Compostela, Spain; 2 CIBER de Fisiopatologia de la Obesidad y Nutricion (CIBERobn), Instituto Salud Carlos III, Santiago de Compostela, Spain; 3 Department of Endocrinology and Nutrition, Hospital del Mar, Barcelona, Spain; 4 Bellon Medical Center, Madrid, Spain; 5 Division of Endocrinology, Complejo Hospitalario Universitario de Ferrol and Coruña University, Ferrol, Spain; 6 Intensive Care Division, Complejo Hospitalario Universitario de Santiago (CHUS), Santiago de Compostela, Spain; 7 Medical Department Pronokal, Pronokal Group, Barcelona, Spain; 8 Center for Research in Metabolic Syndrome, Prediabetes and Diabetes, Fundacion Oftalmologica de Santander (FOSCAL), Floridablanca, Colombia

**Keywords:** Ketogenic diet, Very low-energy diet, Obesity, Ketosis, Acid–base safety, Acidosis

## Abstract

**Background and Aims:**

Very low-calorie ketogenic (VLCK) diets have been consistently shown to be an effective obesity treatment, but the current evidence for its acid-base safety is limited. The aim of the current work was to evaluate the acid-base status of obese patients during the course of a VLCK diet.

**Method:**

Twenty obese participants undertook a VLCK diet for 4 months. Anthropometric and biochemical parameters, and venous blood gases were obtained on four subsequent visits: visit C-1 (baseline); visit C-2, (1-2 months); maximum ketosis; visit C-3 (2-3 months), ketosis declining; and visit C-4 at 4 months, no ketosis. Results were compared with 51 patients that had an episode of diabetic ketoacidosis as well as with a group that underwent a similar VLCK diet in real life conditions of treatment.

**Results:**

Visit C1 blood pH (7.37 ± 0.03); plasma bicarbonate (24.7 ± 2.5 mmol/l); plasma glucose (96.0 ± 11.7 mg/l) as well as anion gap or osmolarity were not statistically modified at four months after a total weight reduction of 20.7 kg in average and were within the normal range throughout the study. Even at the point of maximum ketosis all variables measured were always far from the cut-off points established to diabetic ketoacidosis.

**Conclusion:**

During the course of a VLCK diet there were no clinically or statistically significant changes in glucose, blood pH, anion gap and plasma bicarbonate. Hence the VLCK diet can be considered as a safe nutritional intervention for the treatment of obesity in terms of acid-base equilibrium.

## Introduction

In recent decades the prevalence of obesity has increased considerably worldwide and has now reached epidemic proportions [1–3], which implies potentially serious consequences for the health of the population and the economy [4–7]. Hence, finding effective and safe short-term and long-term treatments for this pathology is a priority. In this sense, very low-calorie-ketogenic (VLCK) diets have consistently shown to be useful tools in the treatment of obesity [8–11]. In fact, our group recently conducted a nutritional intervention clinical trial in which a VLCK diet was shown to be significantly more effective than a standard low-calorie diet after 1 and 2 years of follow-up [10, 11]. Likewise, among various other benefits, a diet-induced weight loss of mainly at the expense of fat-mass and visceral mass, with the preservation of muscle mass and strength has been reported as a clinical advantage of this type of diet [8].

Nonetheless, despite the solid scientific evidence that supports the use of VLCK diets as a useful weight-loss therapy, there is still some fear inherent in their usage because of their mechanism of action [12–14]. This type of diet is characterized by a restriction in carbohydrate and/or calorie intake to the point of inducing a shift in metabolism and the production of plasma ketone bodies [15–18]. Under standard conditions, glucose constitutes the most important substrate for energy utilization, especially in the central nervous system (CNS) which cannot use fatty acids as an energy source [19]. During the period of a VLCK diet, the low-carbohydrate consumption leads to a depletion of the body’s glucose reserves, and therefore the CNS requires an alternative fuel source [20, 21]. Under these circumstances the ketones, which are products of the hepatic oxidation of fatty acids [16], replace the depleted glucose and meet most of the body’s energy requirements [15, 16, 20, 22].

In line with this physiological pathway, individuals with acceptable insulin function who follow low-carbohydrate diets theoretically should experience ketonemia without acidemia, illness or any metabolic complication [23, 24].Indeed, some previous studies have determined that the production of ketone bodies during a VLCK-diet suggests that the diet-induced ketonemia is a well-tolerated process [25–27], even in type 2 diabetic patients [28]. However, the acidity constant of ketones and its implication in the pathophysiology of ketoacidosis in diabetic and alcoholic patients [29–31] have generated debate in terms of the acid–base safety of this type of diets. Despite the clinical importance of elucidating the real effect of the ketogenic diets on the acid–base status in humans, there is however little solid scientific evidence in this regards [24].

As the concept of ketosis is engraved in the mind of endocrinologists as well as in medical personnel managing diabetic patients as an “emergency signal”, to clearly differentiate the medical state of diet-induced ketosis from diabetic ketoacidosis is a must. Therefore, the aim of the current work was to determine the acid–base status of obese patients during the course of a VLCK diet on an outpatient basis for 4 months.

## Materials and methods

### Study design

This study was an open, uncontrolled, nutritional intervention clinical trial conducted for 4 months, and performed in a single center.

### Study population

The patients attending the Obesity Unit at the Complejo Hospitalario Universitario of Santiago de Compostela, Spain, to receive treatment for obesity were consecutively invited to participate in this study.

The inclusion criteria were as follows, age 18 to 65 years, body mass index (BMI) ≥ 30 kg/m^2^, stable body weight in the previous 3 months, desire to lose weight, and a history of failed dietary efforts. The main exclusion criteria were diabetes mellitus, obesity induced by other endocrine disorders or by drugs, and participation in any active weight loss program in the previous 3 months. In addition, those patients with previous bariatric surgery, known or suspected abuse of narcotics or alcohol, severe depression or any other psychiatric disease, severe hepatic insufficiency, any type of renal insufficiency or gouts episodes, nephrolithiasis, neoplasia, previous events of cardiovascular or cerebrovascular disease, uncontrolled hypertension, orthostatic hypotension, and hydroelectrolytic or electrocardiographic alterations, were excluded. Females who were pregnant, breast-feeding, or intending to become pregnant, and those with child-bearing potential and not using adequate contraceptive methods, were also excluded. Apart from having obesity and metabolic syndrome, the participants were generally healthy.

The study protocol was in accordance with the Declaration of Helsinki and was approved by the Ethics Committee for Clinical Research of Galicia, Santiago de Compostela, Spain (registry 2010/119). Participants gave informed consent before any intervention related to the study. Participants received no monetary incentive.

### Nutritional intervention

All the patients followed a VLCK diet according to a commercial weight loss program (PNK method^®^), which includes lifestyle and behavioral modification support. The intervention included an evaluation by the specialist physician conducting the study, an assessment by an expert dietician, and exercise recommendations. This method is based on a high-biological-value protein preparations obtained from cow’s milk, soybeans, avian eggs, green peas and cereals. Each protein preparation contained 15 g protein, 4 g carbohydrates, 3 g fat, and 50 mg docohexaenoic acid, and provided 90–100 kcal [32].

The weight loss program has five steps (Supplementary Fig. 1) and adheres to the most recent EFSA guidelines of 2015 on total carbohydrates intake [33]. The first three steps consist of a VLCK diet (600–800 kcal/day), low in carbohydrates (<50 g daily from vegetables) and lipids (only 10 g of olive oil per day). The amount of high-biological-value proteins ranged between 0.8 and 1.2 g per each kg of ideal bodyweight, to ensure patients were meeting their minimal body requirements and to prevent the loss of lean mass. In step 1, the patients ate high-biological-value protein preparations five times a day, and vegetables with low glycemic indexes. In step 2, one of the protein servings was substituted by a natural protein (e.g., meat or fish) either at lunch or at dinner. In step 3, a second serving of low fat natural protein was substituted for the second serving of biological protein preparation. Throughout these ketogenic phases, supplements of vitamins and minerals, such as K, Na, Mg, Ca, and omega-3 fatty acids, were provided in accordance with international recommendations [34]. These three steps were maintained until the patient lost the target amount of weight, ideally 80%. Hence, the ketogenic steps were variable in time depending on the individual and the weight loss target.

In steps 4 and 5, the ketogenic phases were ended by the physician in charge of the patient based on the amount of weight lost, and the patient started a low-calorie diet (800–1500 kcal/day). At this point, the patients underwent a progressive incorporation of different food groups and participated in a program of alimentary re-education to guarantee the long-term maintenance of the weight loss. The maintenance diet, consisted of an eating plan balanced in carbohydrates, protein, and fat. Depending on the individual the calories consumed ranged between 1500 and 2000 kcal/day, and the target was to maintain the weight lost and promote a healthy life styles.

During this study, the patients followed the different steps of the method until they reach the target weight or up to a maximum of 4 months of follow-up, although patients remained under medical supervision for the months following the trial.

### Schedule of visits

Throughout the study, the patients completed a maximum of 10 visits with the research team (every 15 ± 2 days), of which four were for a complete (C) physical, anthropometric and biochemical assessment, and the remaining visits were to control adherence and to evaluate potential side effects. The four complete visits were made according to the evolution of each patient through the steps of ketosis as follows: visit C-1 (baseline), normal level of ketone bodies; visit C-2, maximum ketosis (approximately 1–2 months of treatment); visit C-3, reduction of ketosis because of partial reintroduction of normal nutrition (2–3 months); visit C-4 at 4 months, no ketosis (Supplementary Fig. 1). The total ketosis state lasted for 60–90 days only. In all the visits, patients received dietary instructions, individual supportive counsel, and encouragement to exercise on a regular basis using a formal exercise program. Additionally, a program of telephone reinforcement calls was instituted, and a phone number was provided to all participants to address any concerns.

### Anthropometric assessment

All anthropometric measurements were undertaken after an overnight fast (8 to 10 h), under resting conditions, in duplicate, and performed by well-trained health workers. Participants bodyweights were measured to the nearest 0.1 kg on the same calibrated electronic device (Seca 220 scale, Medical Resources, EPI Inc OH, USA), in underwear and without shoes. BMI was calculated by dividing body weight in kilograms by the square of height in meters (BMI = weight (kg)/height^2^ (m)).

### Venous blood gases

Peripheral venous blood samples were taken from any easily accessible peripheral vein, although most of the samples were collected from the antecubital vein and were immediately analyzed. A tourniquet was used to facilitate venipuncture, but it was released about 1 min before the sample was drawn to avoid changes induced by local ischemia [35]. The analyzer has several measuring capabilities but only the following parameters were considered for the present study: acidity (pH), partial pressure of CO2 (pCO2), bicarbonate concentration (HCO3–), base excess (BE—amount of H+ required to return blood pH to reference value) and lactic acid. Partial pressure of oxygen is not reported since venous blood gas analyses are not a good reference of oxygenation because oxygen has already been extracted by the tissues by the time the blood reaches the venous circulation. However, this parameter was not considered necessary for the purposes of the present analysis.

We preferred to perform venous blood gases given that arterial punctures are more painful and carry a higher risk of complications compared to venous punctures. Moreover, venous blood gases have proven to be an adequate technique for the diagnosis of disorders in the acid–base balance [36, 37].

### Determination of levels of ketone bodies

Ketosis was determined by measuring ketone bodies, specifically B-hydroxy-butyrate (B-OHB), in capillary blood by using a portable meter (GlucoMen LX Sensor, A. Menarini Diagnostics, Neuss, Germany). As with anthropometric assessments, all the determinations of capillary ketonemia were made after an overnight fast of 8–10 h. These measurements were performed daily by each patient during the entire VLCK diet, and the corresponding values were reviewed on the machine memory by the research team in order to control adherence. Additionally, B-OHB levels were determined at each visit by the physician in charge of the patient. The measurements reported as “low value” (<0.2 mmol/l) by the meter were assumed as to be zero for the purposes of statistical analyses.

### Biochemical parameters

During the study all the patients were strictly monitored with a wide range of biochemical analyses. However, for the purposes of this work only certain values are reported. Sodium, potassium, chloride, glucose, albumin, creatinine and blood urea nitrogen were performed using an automated chemistry analyzer (Dimension EXL with LM Integrated Chemistry System, Siemens Medical Solutions Inc., USA). Insulin and c-peptide were measured by chemiluminescence using ADVIA Centaur (Bayer Diagnostics, Tarrytown, NY, USA). All the biochemical parameters were measured at the four complete visits.

The anion gap was calculated from serum electrolyte measurements in the following manner: anion gap = (sodium) − (chloride + measured bicarbonate). Whereas osmolarity was estimated according to the following formula: osmolarity = (2 × sodium) + (potassium) + (glucose / 18) + (blood urea nitrogen / 2.8). Finally, HOMA-IR was calculated as follows: HOMA-IR = (insulin ×  glucose)/405.

### Reported cases of diet-induced ketoacidosis

To find all the reported cases of ketoacidosis during the course of a ketogenic diet in obese non-diabetic patients we performed a search on the PubMed database using the query “ketoacidosis AND (ketogenic diet OR low carbohydrate diet)”.The search was limited to “English”-language articles, “human” subjects, and publications prior to February, 28, 2017. The eligibility of the studies was assessed by one reviewer.

A total of 344 articles were identified from the initial search in PubMed. After reviewing all the articles and excluding papers about other diseases (e.g., epilepsy and diabetes), and articles without reporting on acid–base disturbances, a total of four cases of ketoacidosis were detected. Subsequently a manual review of the PubMed database was conducted and an additional case was detected, resulting in a total of five cases of ketoacidosis [38–42]. In the present work we will try to describe the possible causes that led to the appearance of ketoacidosis in these non-diabetic patients.

### Diabetic ketoacidosis

Given that the medical state of diet-induced ketosis may be confused with the pathological and life-threatening state of diabetic ketoacidosis, we have included a sub-analysis in which we compare our participants (VLCK diet) with a cohort of patients with diabetic ketoacidosis from another of our centers, with the purpose of finding the possible differences and similarities between both metabolic states. This cohort of patients is formed by all the patients with diabetes of any type that were admitted consecutively to the emergency room of the Hospital del Mar, Barcelona, Spain, with a diagnosis of diabetic ketoacidosis between January 2010 and December 2011.

### Diet-induced ketosis in real life conditions

To show the behavior of diet-induced ketosis in a real-life setting, we have included the results of 460 capillary ketone bodies determinations corresponding to 163 patients during different steps of a VLCK diet according to the same commercial weight loss program (PNK method^®^) that we have used for the present study. All these patients were being treated for being overweight or for obesity in the Centro Medico Bellon, Madrid, Spain.

### Statistical analysis

Continuous variables are presented as mean (standard deviation), whereas categorical variables are presented as frequencies (percentages). All statistical analyses were carried out using Stata statistical software, version 12.0 (Stata, College Station, TX). AP < 0.05 was considered statistically significant. Changes in the different variables of interest from baseline and throughout the study visits were analyzed following a repeated measures design. A repeated measures analysis of variance test was used to evaluate differences between different measurement times, followed by post hoc analysis with Turkey’s adjustment for multiple comparisons. In addition, two-sample equal-variance t-test was used to assess the differences between our study population (VLCK diet) and the cohort of patients with diabetic ketoacidosis.

## Results

### Anthropometrical and biochemical changes during the VLCK diet

A total of 20 obese subjects, 12 females, age from 18 to 58 years (47.2 ± 10.2 yr) completed the study. At baseline (visit C-1), patients had a body weight (bw) of 95.9 ± 16.3 kg and BMI of 35.5 ± 4.4. BMI was weigh (Kg) divided by height in meters squared. Other baseline characteristics and their corresponding changes during the study are presented in Table 1, and have also been previously reported [8].

**Table 1:** Changes in anthropometry, venous blood gases, biochemical parameters and ketone bodies during the study

	VLCK diet			LC diet
	Visit C-1	Visit C-2	Visit C-3	Visit C-4
Diet time (days)		39.2 ± 8.4	89.7 ± 19.1	123.3 ± 17.6
Anthropometry				
Weight (kg)	95.9 ± 16.3	84.2 ± 13.0^a^	76.6 ± 11.1^a,b^	75.1 ± 11.8^a,b^
Weight change (kg)		−11.7 ± 3.7^d^	−19.3 ± 6.4^d^	−20.7 ± 6.9^d^
Weight change (%)		−12.0 ± 2.0^d^	−19.7 ± 4.0^d^	−21.3 ± 4.8^d^
BMI (kg/m^2^)	35.5 ± 4.4	31.2 ± 3.3^a^	28.4 ± 2.6^a,b^	27.8 ± 2.9^a,b^
Venous blood gases				
pH	7.37 ± 0.03	7.37 ± 0.02	7.36 ± 0.02	7.37 ± 0.02
*p*CO_2_ (mmHg)	44.3 ± 6.7	41.6 ± 5.5	42.8 ± 5.4	45.3 ± 4.8
Measured bicarbonate (mmol/l)	24.7 ± 2.5	23.6 ± 2.4	24.1 ± 2.4	25.8 ± 2.0^b,c^
Base excess (mmol/l)	−0.7 ± 1.7	−1.4 ± 1.7	−1.2 ± 1.9	0.2 ± 1.6^b,c^
Lactic acid (mmol/l)	1.5 ± 0.3	1.4 ± 0.2	1.4 ± 0.3	1.4 ± 0.4
Biochemical parameters				
Sodium (mmol/l)	141.0 ± 1.4	142.0 ± 2.0	141.2 ± 1.6	141.5 ± 2.6
Potassium (mmol/l)	4.3 ± 0.2	4.2 ± 0.2	4.2 ± 0.2	4.1 ± 0.3
Chloride (mmol/l)	105.1 ± 1.7	104.8 ± 1.8	105.1 ± 1.6	106.1 ± 2.3^b^
Anion gap	10.9 ± 2.7	13.5 ± 2.2^a^	11.9 ± 2.3	9.5 ± 2.2^b,c^
Osmolarity (mOsm/l)	304.0 ± 5.4	302.0 ± 5.6	303.1 ± 5.0	303.7 ± 6.2
Glucose (mg/dl)	96.0 ± 11.7	78.7 ± 9.5^a^	77.6 ± 8.1^a^	84.8 ± 7.5^a,b,c^
Albumin (gr/dl)	3.8 ± 0.2	4.1 ± 0.1^a^	3.9 ± 0.1^a,b^	3.8 ± 0.2^b^
Creatinine (mg/dl)	0.6 ± 0.1	0.6 ± 0.1	0.6 ± 0.1	0.6 ± 0.1
Blood ureanitrogen (mg/dl)	34.3 ± 10.0	26.3 ± 6.1^a^	34.1 ± 7.8^b^	33.1 ± 8.5^b^
Insulin (mUI/l)	20.4 ± 10.7	8.3 ± 3.4^a^	7.3 ± 2.9^a^	9.2 ± 5.2^a^
C-peptide (ng/ml)	2.2 ± 0.7	1.2 ± 0.4^a^	1.0 ± 0.2^a^	1.3 ± 0.4^a^
HOMA-IR	5.0 ± 2.8	1.5 ± 0.4^a^	1.4 ± 0.6^a^	1.9 ± 1.1^a^
Ketone bodies				
B-hydroxy-butyrate (mmol/l)	0.0 ± 0.1	1.0 ± 0.6^a^	0.7 ± 0.5^a,b^	0.2 ± 0.1^b,c^
Subjects with ketosis (n-%)*	2 (10)	20 (100)	19 (95)	9 (45)

Data are presented as mean ± standard deviation and counts − calculated percentages (*)

Anion gap = (sodium) − (chloride + measured bicarbonate)

Osmolarity = (2 × sodium) + (potassium) + (glucose /18) + (blood urea nitrogen / 2.8)

HOMA-IR = (insulin × glucose)/405

^a^ *P* < 0.05 compared with Visit C-1

^b^ *P * <  0.05 compared with Visit C-2

^c^ *P * <  0.05 compared with Visit C-3 (Repeated measures ANOVA with Tukey’s adjustment for multiple comparisons)

^d^ *P * <  0.05 weight change significantly different from zero (Student’s *t*-test)

**Fig. 1:**
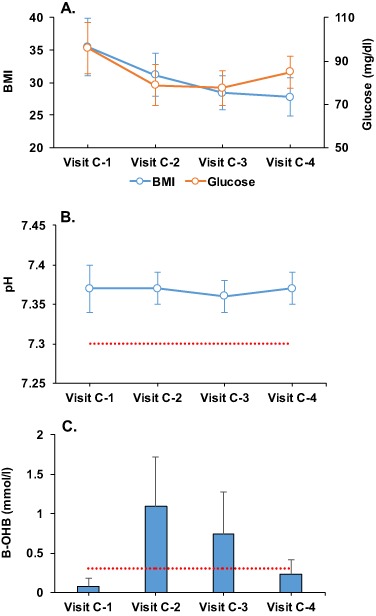
Changes in BMI, glucose, venous blood pH and capillary ketone bodies (B-hydroxy-butyrate) during the study. Data are presented as mean ± standard deviation. *BMI* body mass index. The broken line represents the level at which it is defined the existence of diabetic ketoacidosis (**b**) and ketosis (**c**)

All the patients underwent a total of ten visits, but a complete anthropometrical and biochemical assessment was performed at four of these visits, which were synchronized with the measurements B-OHB levels. Visit C-1 was the baseline visit, before starting the nutritional intervention, with no ketosis (0.0 ± 0.1 mmol/l) and initial weight. Visit C-2 was in accordance with the time of maximum ketosis (1.0 ± 0.6 mmol/l) with 11.7 kg of bw loss. At visit C-3 the patients showed a reduction in levels of B-OHB (0.7 ± 0.5 mmol/l) because of a partial reintroduction of a normal diet, and had a bw loss of 19.3 kg. Finally, at visit C-4 the patients were out of ketosis (0.2 ± 0.1 mmol/l) with a total bw loss of 20.7 kg (Table 1 and Fig. 1). Glucose levels were not significantly modified throughout the study (Fig. 1).

**Fig. 2:**
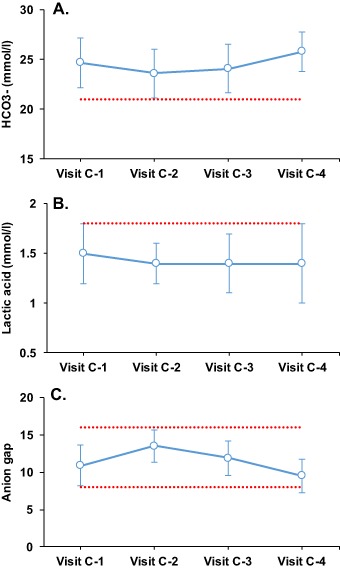
Changes in venous blood gases parameters during the study. Data are presented as mean ± standard deviation. HCO3, measured bicarbonate. The broken line represents the level at which it is defined the existence of hypobicarbonatemia (**a**) and lacticemia (**b**), and the normal range of the anion gap (**c**)

The blood pH was not significantly different from the baseline at any time during the study, and their values were always within the normal range (Table 1 and Fig. 1). At baseline the blood pH level was 7.37 ± 0.03, at visit C-2 after near 40 days of diet and at the point of maximum ketosis the pH was 7.37 ± 0.02, at visit C-3 the venous pH remained unchanged (7.36 ± 0.02), as in the final visit (7.37 ± 0.02). A blood pH level of ≤7.30 is used in the literature to define diabetic ketoacidosis, but in our study no individual had a measurement below this level at any time point (Fig. 1). Metabolic acidosis may also be expressed as a decrease in bicarbonate because it is used to compensate for (buffer) acid metabolites. In the present study, there were also no significant variations in bicarbonate values (mmol/l) during the VLCK diet (24.7 ± 2.5 (baseline), 23.6 ± 2.4 (visit C-2), 24.1 ± 2.4 (visit C-3), and 25.8 ± 2.0 (visit C-4)). It is notable that these values remained unchanged and within the normal ranges even at the visit of the highest level of B-OHB (Table 1 and Fig. 2). Lactic acid did not show significant variations during the study, and the calculated anion gap was always in the reference range (Table 1 and Fig. 2). Importantly, serum albumin levels were within the limits of normality throughout the study. Although there were some differences in electrolytes and blood urea nitrogen over the 4 months of dieting, none of them was considered as clinically relevant (Table 1).

The hypothesis regarding the possible association between ketoacidosis and altered insulin function was assessed by a strict analysis of the glucose metabolism. A considerable improvement in insulin sensitivity was observed in accordance with bw reduction [43] (Table 1). This observation agrees with the idea that in patients with an acceptable insulin function a ketogenic diet induces a well-tolerated ketosis rather than ketoacidosis.

### Diet-induced ketosis vs. diabetic ketoacidosis

To investigate the similarities and differences between the two metabolic states (i.e., diet-induced ketosis and diabetic ketoacidosis), we compared our study sample and a cohort of diabetic patients with ketoacidosis. Blood pH was significantly lower in the diabetic patients with ketoacidosis than in our study sample (7.16 ± 0.12 vs. 7.37 ± 0.02, *P* < 0.001) (Table 2). Serum bicarbonate was also significantly inferior in the cohort of diabetics (12.3 ± 5.7 vs. 23.6 ± 2.4, *P* < 0.001). In addition, there were also significant differences in electrolytes values, including the anion gap which was significantly higher in the cohort of diabetic patients (30.3 ± 7.9 vs. 13.5 ± 2.2, *P* < 0.001). Finally, plasma glucose and B-OHB levels were significantly higher in the diabetic patients, reinforcing the idea that underlying alterations in glucose metabolism play an essential role in the pathogenesis of ketoacidosis.

**Table 2:** Comparison of the biochemical parameters between our study population (very low-calorie-ketogenic diet) and a cohort of patients with diabetic ketoacidosis

	VLCK diet	Diabetic ketoacidosis	*P* value
Number of patients	20	51	
Age (years)	47.2 ± 10.2	39.0 ± 13.5	0.016
Venous blood gases			
pH	7.37 ± 0.02	7.16 ± 0.12	<0.001
Measured bicarbonate (mmol/l)	23.6 ± 2.4	12.3 ± 5.7	<0.001
Base excess (mmol/l)	−1.4 ± 1.7	−18.1 ± 14.8	<0.001
Biochemical parameters			
Sodium (mmol/l)	142.0 ± 2.0	133.8 ± 6.3	<0.001
Potassium (mmol/l)	4.2 ± 0.2	5.0 ± 0.7	<0.001
Chloride (mmol/l)	104.8 ± 1.8	94.9 ± 6.9	<0.001
Anion gap	13.5 ± 2.2	30.3 ± 7.9	<0.001
Glucose (mg/dl)	78.7 ± 9.5	545.5 ± 245.9	<0.001
Ketone bodies			
B-hydroxy-butyrate (mmol/l)	1.0 ± 0.6	5.4 ± 1.2	<0.001

Data are presented as mean ± standard deviation. Data of the VLCK diet group correspond to the visit of maximum ketosis (Visit C-2), whereas the data of patients with diabetic ketoacidosis correspond to the time of hospital admission.

*P* value = two-sample *t*-test with equal variances

### Diet-induced ketosis in real life conditions

**Fig. 3:**
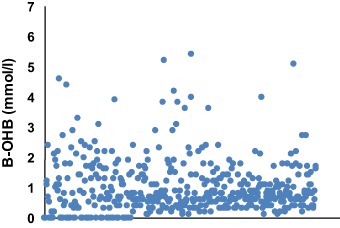
Capillary B-OHB levels during different steps of a very low-calorie-ketogenic diet. There are shown 460 capillary ketone bodies determinations

With the purpose of confirming the moderate production of ketones during the course of a VLCK diet, here we reported several capillary B-OHB determinations during different steps of a VLCK diet corresponding to a cohort of patients undergoing treatment for obesity in a real life setting. Interestingly, none of the patients reached values greater than 6 mmol/l at any point of the diet and the majority of the determinations were less than 3 mmol/l (Fig. 3).

## Discussion

The main findings of this work were as follows: (a) a VLCK diet appears as a safe nutritional intervention, inducing a severe body weight reduction without altering the acid–base balance; (b) No one of the parameters measured, even ketone bodies, reached or even approached the cutoffs commonly accepted for diabetic ketoacidosis; and (c) Results clearly departed from those observed in our patients who had an episode of diabetic ketoacidosis.

Several studies have shown the high value of the VLCK diets as a weight-loss treatment [8–11, 25–27], but its theoretical acid–base safety had not yet to be studied in depth. In patients with near normal or normal insulin function, the plasma B-OHB concentration should reach levels similar to those here reported during the course of a ketogenic diet or fasting because the rate of hepatic ketones bodies synthesis is compensated by the rate of body’s ketones utilization, plus a small degree of ketones loss into the urine [15, 20–22, 24, 44]. Therefore there would be no alteration of the acid–base balance during dieting.

The results of the current study corroborated these hypotheses as no pathological changes in blood pH or plasma bicarbonate were detected after the diet, and the maximum levels of B-OHB were 2.9 mmol/l. In agreement with these results, the majority of the obese non-diabetic patients that were under a VLCK diet in real life conditions also reached B-OHB levels lower than 3.0 mmol/l and no one higher than 6 mmol/l. Notably, in the present study the phase of ketosis was longer than what has been in many other studies, which proves the acid–base safety of this type of diet. The other significant parameters such as glucose, lactic acid, osmolarity and anion gap were not modified from baseline values.

Considering that hepatic generation of ketones is stimulated by the combination of low insulin levels and high glucagon levels (i.e., a low insulin/glucagon ratio) [29, 45] and occurs at rates proportional to the oxidation of fatty acids [16, 17], the maintenance of B-OHB within a normal range and avoiding the development of ketoacidosis during a VLCK diet could be mediated by various metabolic pathways. Initially, the ketones can slow down the release of fatty acids from the adipose tissue by three different mechanisms: stimulation of insulin production despite low plasma glucose levels [46],improving the sensitivity of adipose tissue to the inhibitory effect of insulin on the release of fatty acids [47], and a direct inhibitory effect of lipolysis by the ketones themselves [47]. Moreover, the CNS increases its rate of ketone bodies uptake; because of the decrease in body’s glucose reserves the brain uses ketones as its source of energy [15, 20, 48]. Finally, increased peripheral tissue ketone utilization has also been described [49].

To the best of our knowledge, only one previous study has evaluated acid–base safety during the course of a ketogenic diet [24]. In that previous study, the authors found that a low-carbohydrate ketogenic diet induced a mild compensated metabolic acidosis, unlike our study in which we did not find any pathological variation in the parameters related to acid–base balance. However, that study had some limitations, principally, there were high rates of patients out of ketosis during dieting and measurements were made infrequently. In the present study, during maximum ketosis there was an adherence rate of 100%; additionally, the visits were programmed in accordance with the different stages of ketosis, ensuring an adequate evaluation of the relationship between ketosis and acidosis.

Given the above arguments, it seems reasonable to affirm that diet-induced ketosis and diabetic ketoacidosis are two different processes. Indeed, the most widely used diagnostic criteria for diabetic ketoacidosis include plasma glucose > 250 mg/dl, blood pH < 7.3, serum bicarbonate < 15 mEq/l and a moderate-to-high degree of ketonemia [50], and certainly none of these characteristics was presented in our patients. Likewise, when comparing our study population with the cohort of diabetic patients with ketoacidosis, the metabolic behavior of the two populations was clearly different.

The rationale for the present work was to eliminate the alarm that the word ketosis arise in medical personal. That feeling was also fueled by some reports of hypocaloric diet-induced ketoacidosis, although there were very few [38–42]. A close look to these reports could clarify the issue. Any severe hypocaloric diet must be avoided on a gestational or lactating woman as appears in the case report [42]. The important glucose requirements and tendency to ketosis under lactation may easily explain the negative effects of a very low calorie, high fat diet on this patient on the other hand obesity is not on emergency situation, then any diet can be upheld until the lactation period be finished (41). In the same line the prescription of very low calorie-high fat diets in non obese subjects, i.e., BMI 26.7 with 4 years of hyperproteic [41], or non obese patients with moderate overweight after Ramadan or alcohol intake [38, 40], may explain a severe decompensation; mostly when in one case diet was undertaken after an intercurrent gastrointestinal episode, perhaps moderate pancreatitis [39]. In all the case reported the diet were of very low content in carbohydrates, less than 20 g/day and, high amounts of fat [39] and this reinforce the message that diet should only be prescribed by well trained doctors since diets with less than 20 g of daily carbohydrate consumption, would also contribute to the development of ketoacidosis [38, 41, 42]. On the contrary the Pronokal method followed in the present work is low in carbohydrates (<50 g) and fat and moderately increased in proteins according with international recommendation (see methods section). Under conditions of carbohydrates availability, glycolysis generate citrate which inhibits carnitine palmitoyltransferase complex I, limiting the beta-oxidation of fatty acids and thereby reducing ketogenesis [41]. In addition, dehydration [50] and an enzymatic predisposition to the condition could also be implicated as the causes of ketoacidosis in certain persons [41]. Therefore, an exacerbated production of ketone bodies and the subsequent development of ketoacidosis could be expected to appears in the absence of carbohydrates and a completely insulin-deficient condition in predisposed subjects [44, 51].

In conclusion, this study shows that the VLCK diet is a safe nutritional intervention for the treatment of obesity in terms of the acid–base equilibrium. Apart from ketosis both in this clinical trial and in real life situation all the relevant biochemical parameters were not significantly altered by the VLCK dieting.

## Electronic supplementary material

Supplementary Figure 1

## References

[1] Apovian CM, Aronne LJ, Bessesen DH, McDonnell ME, Murad MH, Pagotto U, Ryan DH, Still CD, Endocrine S (2015). Pharmacological management of obesity: an Endocrine Society clinical practice guideline. J. Clin. Endocrinol. Metab..

[2] Han TS, Correa E, Lean ME, Lee DM, O’Neill TW, Bartfai G, Forti G, Giwercman A, Kula K, Pendleton N, Punab M, Rutter MK, Vanderschueren D, Huhtaniemi IT, Wu FC, Casanueva FF (2017). and the ESG. Changes in prevalence of obesity and high waist circumference over four years across European regions: the European male ageing study (EMAS). Endocrine.

[3] Heymsfield SB, Wadden TA (2017). Mechanisms, pathophysiology, and management of obesity. N. Engl. J. Med..

[4] Crujeiras AB, Cabia B, Carreira MC, Amil M, Cueva J, Andrade S, Seoane LM, Pardo M, Sueiro A, Baltar J, Morais T, Monteiro MP, Lopez-Lopez R, Casanueva FF (2016). Secreted factors derived from obese visceral adipose tissue regulate the expression of breast malignant transformation genes. Int. J. Obes. (Lond)..

[5] Heitmann BL, Erikson H, Ellsinger BM, Mikkelsen KL, Larsson B (2000). Mortality associated with body fat, fat-free mass and body mass index among 60-year-old swedish men-a 22-year follow-up. The study of men born in 1913. Int. J. Obes. Relat. Metab. Disord..

[6] Van Gaal LF, Maggioni AP (2014). Overweight, obesity, and outcomes: fat mass and beyond. Lancet.

[7] Wang YC, McPherson K, Marsh T, Gortmaker SL, Brown M (2011). Health and economic burden of the projected obesity trends in the USA and the UK. Lancet.

[8] Gomez-Arbelaez D, Bellido D, Castro AI, Ordonez-Mayan L, Carreira J, Galban C, Martinez-Olmos MA, Crujeiras AB, Sajoux I, Casanueva FF (2017). Body composition changes after very-low-calorie ketogenic diet in obesity evaluated by 3 standardized methods. J. Clin. Endocrinol. Metab..

[9] Merra G, Gratteri S, De Lorenzo A, Barrucco S, Perrone MA, Avolio E, Bernardini S, Marchetti M, Di Renzo L (2017). Effects of very-low-calorie diet on body composition, metabolic state, and genes expression: a randomized double-blind placebo-controlled trial. Eur. Rev. Med. Pharmacol. Sci..

[10] Moreno B, Bellido D, Sajoux I, Goday A, Saavedra D, Crujeiras AB, Casanueva FF (2014). Comparison of a very low-calorie-ketogenic diet with a standard low-calorie diet in the treatment of obesity. Endocrine.

[11] Moreno B, Crujeiras AB, Bellido D, Sajoux I, Casanueva FF (2016). Obesity treatment by very low-calorie-ketogenic diet at two years: reduction in visceral fat and on the burden of disease. Endocrine.

[12] Frigolet ME, Ramos Barragan VE, Tamez Gonzalez M (2011). Low-carbohydrate diets: a matter of love or hate. Ann. Nutr. Metab..

[13] Paoli A (2014). Ketogenic diet for obesity: friend or foe?. Int. J. Environ. Res. Public Health.

[14] Basciani S, Costantini D, Contini S, Persichetti A, Watanabe M, Mariani S, Lubrano C, Spera G, Lenzi A, Gnessi L (2015). Safety and efficacy of a multiphase dietetic protocol with meal replacements including a step with very low calorie diet. Endocrine.

[15] Cahill GF (2006). Fuel metabolism in starvation. Annu. Rev. Nutr..

[16] McGarry JD, Foster DW (1980). Regulation of hepatic fatty acid oxidation and ketone body production. Annu. Rev. Biochem..

[17] Robinson AM, Williamson DH (1980). Physiological roles of ketone bodies as substrates and signals in mammalian tissues. Physiol. Rev..

[18] Sumithran P, Proietto J (2008). Ketogenic diets for weight loss: a review of their principles, safety and efficacy. Obes. Res. Clin. Pract..

[19] Yang SY, He XY, Schulz H (1987). Fatty acid oxidation in rat brain is limited by the low activity of 3-ketoacyl-coenzyme A thiolase. J. Biol. Chem..

[20] Owen OE, Morgan AP, Kemp HG, Sullivan JM, Herrera MG, Cahill GF (1967). Brain metabolism during fasting. J. Clin. Invest..

[21] Ruderman NB, Ross PS, Berger M, Goodman MN (1974). Regulation of glucose and ketone-body metabolism in brain of anaesthetized rats. Biochem. J..

[22] Owen OE, Felig P, Morgan AP, Wahren J, Cahill GF (1969). Liver and kidney metabolism during prolonged starvation. J. Clin. Invest..

[23] Phinney SD, Bistrian BR, Wolfe RR, Blackburn GL (1983). The human metabolic response to chronic ketosis without caloric restriction: physical and biochemical adaptation. Metabolism.

[24] Yancy WS, Olsen MK, Dudley T, Westman EC (2007). Acid-base analysis of individuals following two weight loss diets. Eur. J. Clin. Nutr..

[25] Brehm BJ, Seeley RJ, Daniels SR, D’Alessio DA (2003). A randomized trial comparing a very low carbohydrate diet and a calorie-restricted low fat diet on body weight and cardiovascular risk factors in healthy women. J. Clin. Endocrinol. Metab..

[26] Brehm BJ, Spang SE, Lattin BL, Seeley RJ, Daniels SR, D’Alessio DA (2005). The role of energy expenditure in the differential weight loss in obese women on low-fat and low-carbohydrate diets. J. Clin. Endocrinol. Metab..

[27] Meckling KA, O’Sullivan C, Saari D (2004). Comparison of a low-fat diet to a low-carbohydrate diet on weight loss, body composition, and risk factors for diabetes and cardiovascular disease in free-living, overweight men and women. J. Clin. Endocrinol. Metab..

[28] Goday A, Bellido D, Sajoux I, Crujeiras AB, Burguera B, Garcia-Luna PP, Oleaga A, Moreno B, Casanueva FF (2016). Short-term safety, tolerability and efficacy of a very low-calorie-ketogenic diet interventional weight loss program versus hypocaloric diet in patients with type 2 diabetes mellitus. Nutr. Diabetes.

[29] R BD, P TW (2001). Clinical physiology of acid-base and electrolyte disorders.

[30] Jenkins DW, Eckle RE, Craig JW (1971). Alcoholic ketoacidosis. JAMA.

[31] Levy LJ, Duga J, Girgis M, Gordon EE (1973). Ketoacidosis associated with alcoholism in nondiabetic subjects. Ann. Intern. Med..

[32] Dieta Metodo Pronokal para perder peso y mantenerlo (2015), http://www.pronokal.com/esp/. Accessed 10 Jan 2015

[33] EFSA Panel on Dietetic Products, Nutrition and Allergies (NDA (2015). Scientific opinion on the essential composition of total diet replacements for weight control. EFSA J..

[34] SCOOP-VLCKD task 7.3. *Reports on tasks for scientific cooperation. Collection of data on products intendend for use in very-low-calorie-diets* (European Comission, Brussels, 2002)

[35] Cengiz M, Ulker P, Meiselman HJ, Baskurt OK (2009). Influence of tourniquet application on venous blood sampling for serum chemistry, hematological parameters, leukocyte activation and erythrocyte mechanical properties. Clin. Chem. Lab. Med..

[36] Gokel Y, Paydas S, Koseoglu Z, Alparslan N, Seydaoglu G (2000). Comparison of blood gas and acid-base measurements in arterial and venous blood samples in patients with uremic acidosis and diabetic ketoacidosis in the emergency room. Am. J. Nephrol..

[37] Walkey AJ, Farber HW, O’Donnell C, Cabral H, Eagan JS, Philippides GJ (2010). The accuracy of the central venous blood gas for acid-base monitoring. J. Intensive Care Med..

[38] Chalasani S, Fischer J (2008). South Beach Diet associated ketoacidosis: a case report. J. Med. Case Rep..

[39] Chen TY, Smith W, Rosenstock JL, Lessnau KD (2006). A life-threatening complication of Atkins diet. Lancet.

[40] Freeman TF, Willis B, Krywko DM (2014). Acute intractable vomiting and severe ketoacidosis secondary to the Dukan Diet(c). J. Emerg. Med..

[41] Shah P, Isley WL (2006). Ketoacidosis during a low-carbohydrate diet. N. Engl. J. Med..

[42] von Geijer L, Ekelund M (2015). Ketoacidosis associated with low-carbohydrate diet in a non-diabetic lactating woman: a case report. J. Med. Case Rep..

[43] A.B. Crujeiras, D. Gomez-Arbelaez, M.A. Zulet, M.C. Carreira, I. Sajoux, D. de Luis, A.I. Castro, J. Baltar, I. Baamonde, A. Sueiro, M. Macias-Gonzalez, D. Bellido, F.J. Tinahones, J.A. Martinez, F.F. Casanueva, Plasma FGF21 levels in obese patients undergoing energy-restricted diets or bariatric surgery: a marker of metabolic stress? Int. J. Obes. (Lond). (2017). https://doi.org/10.1038/ijo.2017.138 10.1038/ijo.2017.138 28588304

[44] Yancy WS, Olsen MK, Guyton JR, Bakst RP, Westman EC (2004). A low-carbohydrate, ketogenic diet versus a low-fat diet to treat obesity and hyperlipidemia: a randomized, controlled trial. Ann. Intern. Med..

[45] Miles JM, Haymond MW, Nissen SL, Gerich JE (1983). Effects of free fatty acid availability, glucagon excess, and insulin deficiency on ketone body production in postabsorptive man. J. Clin. Invest..

[46] Madison LL, Mebane D, Unger RH, Lochner A (1964). The hypoglycemic action of ketones. Ii. Evidence for a stimulatory feedback of ketones on the pancreatic beta cells. J. Clin. Invest..

[47] Balasse EO, Fery F (1989). Ketone body production and disposal: effects of fasting, diabetes, and exercise. Diabetes Metab. Rev..

[48] Reichard GA, Owen OE, Haff AC, Paul P, Bortz WM (1974). Ketone-body production and oxidation in fasting obese humans. J. Clin. Invest..

[49] Owen OE, Reichard GA (1971). Human forearm metabolism during progressive starvation. J. Clin. Invest..

[50] Kitabchi AE, Umpierrez GE, Murphy MB, Kreisberg RA (2006). Hyperglycemic crises in adult patients with diabetes: a consensus statement from the American Diabetes Association. Diabetes Care.

[51] Hayami T, Kato Y, Kamiya H, Kondo M, Naito E, Sugiura Y, Kojima C, Sato S, Yamada Y, Kasagi R, Ando T, Noda S, Nakai H, Takada E, Asano E, Motegi M, Watarai A, Kato K, Nakamura J (2015). Case of ketoacidosis by a sodium-glucose cotransporter 2 inhibitor in a diabetic patient with a low-carbohydrate diet. J. Diabetes Investig..

