# Supplementary material for: Acid–base safety during the course of a very low-calorie-ketogenic diet
Source: Endocrine. 2017 Sep 15;58(1):81–90. doi: 10.1007/s12020-017-1405-3 (PMC5608861; doi:10.1007/s12020-017-1405-3)
Supplement: Supplementary file 1 — Supplementary Figure 1 [file 12020_2017_1405_MOESM1_ESM.docx]

**Supplementary figure 1.** Nutritional intervention program and schedule of visits.

* Visit C-4 was performed at the end of the study according to each case, once the patient achieved the target weight or maximum at 4 months of follow-up.
